# Investigation of Psychophysiological and Subjective Effects of Long Working Hours – Do Age and Hearing Impairment Matter?

**DOI:** 10.3389/fpsyg.2017.02167

**Published:** 2018-01-12

**Authors:** Verena Wagner-Hartl, K. Wolfgang Kallus

**Affiliations:** ^1^Department of Psychology, University of Graz, Graz, Austria; ^2^Faculty Industrial Technologies, Furtwangen University, Campus Tuttlingen, Tuttlingen, Germany

**Keywords:** long working hours, age, hearing impairment, cortisol, psychophysiology

## Abstract

Following current prognosis, demographic development raises expectations of an aging of the working population. Therefore, keeping employees healthy and strengthening their ability to work, becomes more and more important. When employees become older, dealing with age-related impairments of sensory functions, such as hearing impairment, is a central issue. Recent evidence suggests that negative effects that are associated with reduced hearing can have a strong impact at work. Especially under exhausting working situations such as working overtime hours, age and hearing impairment might influence employees’ well-being. Until now, neither the problem of aged workers and long working hours, nor the problem of hearing impairment and prolonged working time has been addressed explicitly. Therefore, a laboratory study was examined to answer the research question: Do age and hearing impairment have an impact on psychophysiological and subjective effects of long working hours. In total, 51 white-collar workers, aged between 24 and 63 years, participated in the laboratory study. The results show no significant effects for age and hearing impairment on the intensity of subjective consequences (perceived recovery and fatigue, subjective emotional well-being and physical symptoms) of long working hours. However, the psychophysiological response (the saliva cortisol level) to long working hours differs significantly between hearing impaired and normal hearing employees. Interestingly, the results suggest that from a psychophysiological point of view long working hours were more demanding for normal hearing employees.

## Introduction

Until now, neither the problem of aged workers and long working hours, nor the problem of employees suffering from hearing impairment and prolonged working time has been addressed explicitly in a psychophysiological approach. Moreover, hearing impairment is one of the prominent symptoms of increasing age. The current paper addresses this interplay in a laboratory study.

### Age and Age-Related Impairments

Demographic development raises expectations of an aging of the working population in the next years (e.g., World Health Organization [WHO], 1993; Statistik Austria, 2014), making it more and more important to keep employees healthy and strengthen their ability to work until they reach retirement-age. Demographic changes also require the working world to adapt to fewer available younger professionals; therefore elderly skilled employees have to be involved more in the working process (Müller, 2011). When employees get older, dealing with age-related impairments of sensory functions such as hearing impairment or vision-related impairments, is a central issue. While vision-related impairments are often corrected and the wearing of glasses is not a problem in society, unfortunately the same is not true for hearing impairment. People with hearing impairments often do not own a hearing aid. Reasons for this are for example that they feel that they hear well enough in most situations, hearing aids are uncomfortable or won’t help them and that they would be embarrassed to wear hearing aids or they have not yet tested their hearing (Hougaard and Ruf, 2011). In addition, it also appears that, even if a hearing impaired person owns a hearing aid, hearing aids are sometimes not used regularly by their owners. So far, however, there has been little discussion about dealing with hearing impairment in the working world.

World Health Organization [WHO] (1993) defines aging workers as employees who are 45 years and older. According to World Health Organization [WHO] (2015a) data, over 5% of the world’s population (360 million people) has disabling hearing loss, which has been supported by a recent German study (Gablenz et al., 2017). In addition, the World Health Organization [WHO] (2015b) estimates that due to unsafe listening practices or rather unsafe or damaging levels of sounds (e.g., while listening to their audio devices, visiting clubs, bars or discotheques), 1.1 billion young people could be at risk of hearing loss. Considering this, research regarding reduced hearing capacity while people are employed or capable of employment becomes increasingly important. Hearing impairment has a significant impact on the quality of life of the person and was also found to be associated with reduced subjective emotional well-being and increased symptoms of depression (Scherer and Frisina, 1998; National Academy on an Aging Society, 1999; Mathers et al., 2000; Arlinger, 2003; Dalton et al., 2003; Monzani et al., 2008; Hawkins et al., 2012; Heffernan et al., 2016). Following Hawkins et al. (2012), self-reported hearing impairment influences not only the quality of life negatively, furthermore, the negative influence of hearing impairment seems to be stronger than other chronic medical problems like heart problems, diabetes, hypertension, or arthritis, which may also have an influence on early retirement. This view is supported by the National Academy on an Aging Society (1999) that reported a retirement rate of 18% for hearing-impaired, and a retirement rate of 12% for normal hearing American employees aged between 51 and 61 years. Analyses of data of the whole United States-working population have shown that 75% of the normal hearing compared to 67% of the hearing impaired working-age population was employed. However, a simple causal interpretation of these data is not possible.

Apart from the previously discussed impact on the quality of life of the concerned person itself, uncorrected reduced hearing also influences all persons that want to communicate with persons with hearing impairment (Jennings and Shaw, 2008; Scarincia et al., 2009; Lemke and Scherpiet, 2015). One can easily imagine that this can have a strong impact at work. In addition, previous studies reported negative effects of hearing impairment on (job) performance caused by an impairment of speech comprehension, memory performance and selective attention (Boxtel et al., 2000; Pearman et al., 2000; Neijenhuis et al., 2004; Baskent et al., 2010; Lin et al., 2011; Rönnberg et al., 2011; Rudner et al., 2011; Nachtegaal et al., 2012; Lemke and Scherpiet, 2015; Wingfield et al., 2015; Frtusova and Phillips, 2016; Mudar and Husain, 2016). Furthermore, the information processing of hearing impaired is associated with significantly increased effort (Rabbitt, 1968; Kahneman, 1973; McCoy et al., 2005). To explain these findings, the so-called effortfulness hypothesis is used: Hence, to achieve the same perceptual performance as a normal hearing person, a person with hearing impairment must expend a higher performance effort (or more cognitive resources). This is followed by lower available process resources to decode the content and transfer it to short- and long-term memory. The question arises whether this supposed increase in performance effort of hearing impaired has an influence on their well-being or perceived exhaustion during working hours, especially under exhausting working situations such as working overtime hours.

### Long Working Hours

According to article 2 of the Directive 2003/88/EC of the European Parliament and of the Council of 4 November 2003 concerning certain aspects of the organization of working time (European Parliament and Council of the European Union, 2003; p. L299/10), “working time” is defined as “any period during which the worker is working, at the employer’s disposal and carrying out his activity or duties, in accordance with national laws and/or practice.” “Rest period” is defined as “any period which is not working time.” Generally, occupational scientists define “long working hours” as working hours that are beyond normal weekly hours of work. However, no consistent number of a defined limit of weekly hours can be found in literature beyond which additional hours are considered as long working hours. For example, Caruso et al. (2004) define long working hours as more than 40 working hours a week, whereas Dex et al. (1995) define them as more than 60 h of working. According to article 6 “Maximum weekly working time” of the Directive 2003/88/EC” (European Parliament and Council of the European Union, 2003; p. L299/11), “the average working time for each seven-day period, including overtime, does not exceed 48-hours” [article 6 (b)]. Therefore, this measure is also often used (e.g., Beswick and White, 2003; Kodz et al., 2003). An exact limit for working hours of a working day is not defined in the European Directive, however, a recovery time of 11 h a day is prescribed. Following the European Foundation for the Improvement of Living and Working Conditions (Eurofound, 2016) “overtime” is defined as “time worked in addition to hours worked during normal periods of work, and generally paid at higher rates than normal rates” (p. 5). Following this definition the normal weekly working hours have to be taken into account which can vary in different countries, sectors, and/or organizations (Eurofound, 2016). Also, overtime is not always paid or compensated by compensatory time off. In the United States, a differentiation between “exempt employees” and “non-exempt employees” do exist (Doyle, 2017). Following Doyle (2017) “the term ‘exempt’ means exempt from being paid overtime.” Following the Overtime Pay Requirements of the Fair Labor Standards Act (FLSA; U.S. Department of Labor: Wage and Hour Division, 2012), “FLSA requires employers to pay covered non-exempt employees at least the federal minimum wage and overtime pay for all hours worked over 40 in a work week.” An employee’s workweek is defined as “a fixed and regularly recurring period of 168-hours – seven consecutive 24-hour periods” (U.S. Department of Labor: Wage and Hour Division, 2008; p. 1). Following Dahlgren et al. (2006), long working hours are prevalent within today’s working world, which has been supported by the results of the Foundation’s Third European Survey on Working Conditions (15 EU Member States; Boisard et al., 2003) which showed that at least 17% of the full-time employees are affected by long working hours, i.e., worked more than 45 h a week.

Previous research has shown that the organization of working time has not only an effect on health and social contacts/the social environment, but is also important from an economic point of view. For example, Nachreiner (2002) concludes that the risk of accidents increases exponentially after the seventh and eighth hour of overtime. This in turn results in increased subsequent costs such as remuneration in the event of sickness or increased insurance premiums. Furthermore, overtime increases the error rate, which from a long-term perspective, leads to an increase in economic costs. Other research groups also reported a negative impact on work performance and safety at work (e.g., Dembe et al., 2005; Folkard and Lombardi, 2006; Knauth, 2007). However, there are also studies that could not demonstrate a correlation between longer working hours and an increased risk of accidents (e.g., Trimpop et al., 2000; Akerstedt et al., 2002). Following Caruso’s (2006) summary long working hours have direct impact on health and safety of employees. For example, long working hours are associated with insufficient sleep, as well as general fatigue. Furthermore, employees with long working hours show poorer performance in cognitive tasks, concentration and attention, and additionally, long working hours lead to unhealthy eating habits and a higher risk of long-term illnesses and injuries. On the other hand, not only the employees themselves are affected by long working hours, but also their family environment (Krenn and Hermann, 2004; Geurts et al., 2009). In addition to a subjectively perceived poorer well-being (Caruso et al., 2004), long working hours can have an impact on an increased risk of cardiovascular symptoms (Buell and Breslow, 1960; Uehata, 1991; Liu and Tanaka, 2002) and influence blood pressure negatively (elevated blood pressure through to hypertension; Iwasaki et al., 1998; Park et al., 2001; Rau and Triemer, 2004; Su et al., 2008). In addition, Park et al. (2001) showed an association with lowered heart rate variability. Also, diseases of the musculoskeletal system increase with longer working hours (Raediker et al., 2006; Trinkoff et al., 2006). Furthermore, a relationship between metabolic disorders such as diabetes mellitus or the metabolic syndrome and long working hours was shown (Sparks et al., 1997; Spurgeon et al., 1997; Violanti et al., 2009). The question arises whether a critical interplay between age and age-related impairments like hearing impairment and effects of long working hours do exist. Therefore, age differences regarding long working hours are of importance. Here, a trend reversal seems to take place. Whereas in 1979, in the United States the group of employees with the longest working hours were between 25 and 34 years old and employees aged between 55 and 64 years worked far less, this trend had been reversed in 2006 (Kuhn and Lozano, 2006). Very similar conclusions were shown by Beswick and White (2003) for working hours in the United Kingdom, and the results of the Foundation’s Third European Survey on Working Conditions (15 EU Member States; Boisard et al., 2003) additionally indicate that older employees do not work shorter hours than younger employees. Considering all of this evidence, it seems that studies with an age-specific point of view on the effect of long working hours, becomes more and more important. Especially, due to the expectation that the occurrence of aftereffects of long working hours will manifest themselves as problems with advanced age. For this reason, our study focuses not only on the impact of reduced hearing capacity but also on age.

### Recovery

In addition to previously described general effects of age, age related impairments and long working hours, also the interplay with employees’ recovery, is of interest. Following Sonnentag et al. (2008, p. 675) “recovery is an important concept in the context of job stress and strain.” Recovery is required in order to compensate negative consequences of strain, such as mental fatigue, and to restore conditions to achieve optimum performance (Allmer, 1996). Kallus and Erdmann (1994) define recovery as dynamic psychophysical process. This includes basic biological regulation processes at different physiological levels as well as mental regulation and control processes, up to complex emotions, cognitions, actions, and social interactions. Following Kellmann and Kallus (2000, p. 210) recovery is characterized as follows: “recovery is a process in time, is related to the type of and duration of stress, depends on a reduction of, a change of, or a break from stress, is individually specific and depends on individual appraisal, ends when the psychophysical state of restored efficiency and homeostatic balance is reached, includes purposeful action (active recovery), as well as automated psychological and biological processes restoring the initial state (passive recovery) and can be described on various levels (e.g., physiological level, psychological level, social level, socio-cultural level, environmental level). Furthermore, recovery processes can be displayed in various organismic subsystems, various sub-processes of recovery can be dissociated and recovery is closely tied to boundary conditions (e.g., sleep, social contact, etc.).” Kallus (2016, p. 42) suggests that these characteristics show “that recovery is much more than eliminating fatigue or restarting the system.”

According to Nachtegaal et al. (2009) there is a significant correlation between hearing status and need for recovery after work in a way that a higher need of recovery after work is reported by people with poorer than by people with better hearing status. Furthermore, older employees seem to have a stronger need of recovery than younger employees (e.g., Jansen et al., 2002; Kiss et al., 2008). In addition, Sonnentag and Zijlstra (2006) reported a positive relationship between the amount of overtime work and the need for recovery. From our point of view, there is a gap in literature regarding the combination of these variables and their influence on employees’ recovery and well-being. Our study addresses this gap in literature and therefore focusses on hearing capacity and age under exhausting working situations such as working overtime hour’s, respectively, long working hours.

### Endocrine Stress Parameters

As mentioned before, long working hours can provoke stress. On the one hand, stress can be assessed subjectively (e.g., questionnaires). On the other hand, psychophysiological parameters, such as cardiovascular activity and responses of the endocrine system are able to show stress or rather arousal of an individual from an objective point of view. Regarding the endocrine system, mental, emotional, and physical stress can lead to an increase in cortisol levels within minutes. This is one of the reasons why the psychophysiological parameter cortisol is applied as one of the most important stress hormones besides the catecholamines noradrenaline and adrenaline (Kirschbaum, 1991). Cortisol is controlled by the hypothalamic-pituitary-adrenal axis (HPA axis). If the HPA axis is dysregulated, a hyper- or hyposecretion of cortisol is possible (Pruessner et al., 1997). Cortisol is a steroid hormone produced by the adrenal cortex and affects many physiological systems. It increases the carbohydrate-, fat-, and protein-metabolism and enables a rapid physical performance enhancement and, in addition, affects immune functions. Thus, the organism is able to adequately respond to stress (Selye, 1936). Cortisol is measured at different measurement times in the presented study (see Materials and Methods section). Following a circadian rhythm, the cortisol level is typically high in the morning after waking up, showing an increase by approximately 50–60% in the first 30–45 min after awakening, and decreases rapidly in the first hours thereafter. During the day the cortisol level decreases slowly until it reaches its lowest level around midnight (Pruessner et al., 1997; Wust et al., 2000). The circadian rhythm is very stable over age (healthy persons). Adam et al. (2006) reported no significant effects of age on the wakeup cortisol level.

Jahncke and Halin (2012) used salivary cortisol to investigate differences between hearing impaired and normal hearing participants during a simulated open-plan office working situation. The results show a tendency toward higher stress levels during noise exposure of 60 L_Aeq_, for hearing impaired compared to normal hearing participants. The authors assume that this effect is possibly caused by the fact that hearing impaired are being more noise sensitive and distracted by noise than normal hearing individuals. Controversial results for the relationship between (long) working hours and cortisol levels are shown in literature. Dahlgren et al. (2006) compared a normal working week (8 h/day / 40-h workweek) to a week with long working hours (12 h/day / 60 h/week). No significant main effect of overtime was shown for the salivary cortisol data of the participants but a trend toward an interaction effect for the morning values: An increase at the end of the working week with long working hours compared to the normal working week was reported. Due to the relatively small sample size (18 office workers) the authors suggest to interpret these data with caution. On the other hand, Persson et al. (2003) were not able to show a significant association between working hours and salivary cortisol (two working-hour groups: 8 h/5 days, i.e., 40 h a week; 12 h/7 days, i.e., 84 h a week), which was confirmed in a longitudinal study by Steptoe et al. (1998). Further, Marchand et al. (2012) suggest that the effect of working hours on the cortisol level is non-linear and therefore only becomes visible after a time period of more than two working days. Besides this, a negative correlation between job strain and cortisol occurred in the study of Steptoe et al. (1998). However, Steptoe et al. (2000) showed a higher cortisol level of people that experienced a high-level of job strain compared to people that experienced low job strain. This is in line with other research groups that showed a correlation between workload and cortisol awakening response (CAR) and between workload and a stronger increase of the cortisol level after awakening (e.g., Kunz-Ebrecht et al., 2004; Eller et al., 2006). To sum it up, controversial results for the relationship between working hours and cortisol level are shown in literature, while there is more consensus on the relationship between job strain, workload, and cortisol level.

Whereas effects of gender, smoking status, alcohol consumption or job support were often investigated in the different studies, the combined effects of long working hours, age and hearing impairment were not investigated as important variables – at least not in a psychophysiological approach. If age was mentioned in the studies, the authors report that age was controlled within their samples. Our study extends past research on long working hours by including age as variable of interest. Therefore, the aim of the study presented in this paper was to examine if age and hearing impairment, do have an impact on psychophysiological and subjective effects of long working hours. Accordingly, the following research question should be answered: Do age and hearing impairment have an impact on psychophysiological and subjective effects of long working hours?

## Materials and Methods

A laboratory study with repeated measurement design was chosen. The study was conducted in an experimental laboratory of the department of psychology at the University of Graz, Austria.

### Participants

In total 61 employees (white-collar workers) participated in the laboratory study. Salivary cortisol measurements of 51 of them were available. Loss of salivary cortisol data of 10 participants was due to not analyzable samples or the non-willingness to participate in this psychophysiological measurement. Therefore, for analyses presented within this paper, the final sample consists of 51 white-collar workers, aged between 24 and 63 years (*M* = 39.69, *SD* = 11.62). Following the definition of World Health Organization [WHO] (1993) whereas aging workers are defined as workers who are aged 45 years and older, 22 participants (43.14%) belong to the group of aging workers or older workers. 50.98% of the participants were female and 49.02% were male. All of them performed their work primarily in office workplaces. 7.84% of the participants were self-employed, 21.57% have a leadership position. 54.90% of the participants reported an average overtime of 1–5 h/week in the last 3 months before they participated in the study, 25.50% of 6–10 h/week, 9.80% of 11 and more hours per week and 9.80% did not work overtime hours in the last 3 months. None of the participants owns a hearing aid. Participants’ hearing abilities were screened by audiometry. Their hearing loss measured by audiometry ranged for the worse hearing ear from 2.50 to 46.25 pure tone average (PTA) dB HL (*M* = 11.47, *SD* = 7.06). The criterion to be included in the hearing impaired group was a worse ear hearing loss of 15 dB or more on a minimum of two out of the four (speech relevant) frequencies 0.5, 1, 2, and 4 kHz. Following the criterion, 21 participants (female: 12, male: 9; age: *M* = 45.67, *SD* = 12.12) were included in the hearing impaired group, 30 participants (female: 14, male: 16; age: *M* = 35.50, *SD* = 9.34) were included in the normal hearing group. The unequal distribution regarding age of the two groups, *t*(49) = -3.38, *p* = 0.001, can be explained by the natural aging process of auditory functions. To control possible effects of the commute of the participants from their workplace to the experimental laboratory, a baseline measurement of all study variables was performed at the beginning of the experimental session. Regarding the study variables, no significant differences at the baseline measurement were found for the two different hearing groups. Therefore, the effects can be attributed to the study manipulation. In addition the hearing groups do not differ significantly regarding average overtime per week in the last 3 months nor regarding the number of overtime hours in the current working week in which they participated in the study.

The participants were recruited via the homepage of the University of Graz, short communications in regional newspapers and notices that were posted at notice boards in different companies, supermarkets, universities, medical practices of otolaryngologist and hearing aid acousticians. All participants received 65 Euro to refund their transportation costs and as incentive for their participation. Informed written consent was obtained from all participants.

### Study Design, Materials, and Procedure

To answer the research question if age and hearing impairment do have an impact on psychophysiological and subjective effects of long working hours, a repeated measurement design was chosen for the laboratory study. Hearing impairment [normal hearing employees, hearing impaired employees (worse ear hearing loss of 15 dB or more on a minimum of two out of the four (speech relevant) frequencies 0.5, 1, 2, and 4 kHz)] was defined as independent variable and age of the participants was included as covariate in the analyses. Five different dependent variables (DV) were measured: DV1: perceived recovery, DV2: perceived fatigue, DV3: subjective emotional well-being, DV4: subjective physical symptoms, DV5: cortisol level.

Time of measurement was used as repeated measurement factor. Overall, each study participant participated in the study for two consecutive days. To participate in the study, each study participant came to our laboratory of the University of Graz directly after his/her regular working hours. On both days the participants had to work in the study (performance tests, etc. – Vienna Test System, Schuhfried GmbH) at least three additional hours to simulate a situation of long working hours (day 1: three to max. five additional hours; day 2: three additional hours). The examination procedure is based on the “Grazer fatigue paradigm” (Deixelberger-Fritz et al., 2003), which is well-established for experimental studies of stress, and especially fatigue. With the choice of a long period of execution and carrying out the investigation after a normal working day of the participants, the approach emphasizes the results of Healy et al. (2004). These results suggest that a reliable measurement of fatigue may only be possible after a working duration of at least 1–2 h on top of the usual working hours per day.

Two measurement times were analyzed for all dependent variables (subjective measures and saliva cortisol level): (1) at the end of the study session of the first day of the laboratory study, and (2) at the end of the study session of the second day of the laboratory study. In addition, a third measurement time was available for saliva cortisol level: the waking cortisol level on the second day of the study.

#### Pure-Tone Audiometry

Pure-tone audiometry was conducted in the beginning of the first study session, using a standard Audiometer (Micromate 304, Madsen Electronics). Following the WHO (Mathers et al., 2000) and the European Working Group on Genetics of Hearing Impairment [EUWG] (1996) hearing loss was measured by audiometry and calculated on the basis of the pure-tone average (PTA) of hearing thresholds at 0.5, 1, 2, and 4 kHz.

#### Saliva Cortisol

To measure cortisol in saliva, saliva samples were obtained with Salivette tubes (Sarstedt) at three measurement times during the study: (1) at the end of the study session of the first day of the laboratory study, (2) the waking cortisol level on the second day of the study and (3) at the end of the study session of the second day of the study. Measurement time (1) and (3) were instructed by the investigator. Only measurement time (2), the waking cortisol level had to be carried out independently by the study participants. The participants were instructed to take the saliva sample 30 min after waking up in the morning and not to brush their teeth, eat, drink, smoke, or do physically demanding activities before providing the saliva sample. The exact time had to be recorded in a protocol. The saliva samples were analyzed at the Technical University of Dresden (Department of Psychology, Biopsychology, Prof. Dr. Kirschbaum) by a professional blind to the experimental conditions. There, the samples were centrifuged for 5 min at a rotation speed of 3,000 rotations/min. The cortisol concentration was measured using cortisol luminescence immunoassay (CLIA) with a high sensitivity of 0.16 ng/ml.

#### Subjective Emotional Well-being

For the measurement of the current subjective emotional well-being a category adjective checklist (German version) consisting of 24 items (BSKE-24-ak) from Janke et al. (1986) was used. The BSKE is based on the German Adjective Checklist EWL (Janke and Debus, 1978). It assesses the current emotional state multidimensional. The eight different sub-dimensions of the BSKE are: balance, lifted mood, activation, excitement, irritability, anxiety/sadness, de-activation, and extraversion. Responses are based on a seven-point scale ranging from 0 (not at all) to 6 (most intensive). Reliabilities are given as 0.70 to 0.90 (Janke and Debus, 1978). Example item: “*Feeling of emotional well-being (e.g., pleasant, satisfied) … 0 (not at all) to 6 (most intensive).”* Two measurement times were analyzed: (1) at the end of the study session of the first day of the study, and (2) at the end of the study session of the second day of the laboratory study.

#### Subjective Evaluation of Perceived Recovery

For the measurement of perceived recovery, the German version of the scale for perceived recovery (SwE) from Kallus and Eibel (2007) was used. Responses are based on 7 descriptive categories and 51 fine adjustments from 0 (not at all recovered) to 51 (extremely strong recovered) following the method of the category subdivision approach (Heller, 1985). Participants have to assess their current perceived recovery by following a two-step procedure: First participants have to scale their perceived recovery in one of seven descriptive categories (“not at all recovered” to “extremely strong recovered”) and afterward, they have to select one out of 10 levels within the initially selected descriptive category (except for the two extrema not at all/extremely strong recovered, where only one level is available). Two measurement times were analyzed: (1) at the end of the study session of the first day of the study, and (2) at the end of the study session of the second day of the laboratory study.

#### Subjective Evaluation of Perceived Fatigue

For the measurement of perceived fatigue, the German version of the scale for perceived fatigue (SwM) from Kallus and Eibel (2008) was used. Responses are based on 7 descriptive categories and 51 fine adjustments from 0 (not at all fatigued) to 51 (extremely strong fatigued) following the method of the category subdivision approach (Heller, 1985). Participants have to assess their current perceived fatigue by following a two-step procedure: First participants have to scale their perceived fatigue in one of seven descriptive categories (“not at all fatigued” to “extremely strong fatigued”) and afterward, they have to select one out of ten levels within the initially selected descriptive category (except for the two extrema not at all/extremely strong fatigued, were only one level is available). Two measurement times were analyzed: (1) at the end of the study session of the first day of the study, and (2) at the end of the study session of the second day of the laboratory study.

#### Subjective Physical Symptoms

For the measurement of the current subjective physical symptoms the 24-item German version of the multidimensional physical symptom list (MKSL-24-ak) from Erdmann and Janke (1994) was used. The seven different sub-dimensions of the MKSL are: pain; nausea, cholinergic symptoms; vegetative symptoms; adrenergic symptoms; general physical relaxation; palpitations; flushing, sensation of heat. Responses are based on a seven-point scale ranging from 0 (not at all) to 6 (most intensive). Reliabilities are given as 0.30 to 0.70 (Krejcza, 2006). Example item: “*Feeling physical weakness or physical exhaustion … 0 (not at all) to 6 (most intensive).”* Two measurement times were analyzed: (1) at the end of the study session of the first day of the study, and (2) at the end of the study session of the second day of the laboratory study.

### Statistical Analyses

The statistical analyses of the data were conducted using the software SPSS for Windows. MANCOVAs and ANCOVAs with repeated measures were used as statistical procedure. The analyses were based on a significance level of 5%.

## Results

### Subjective Emotional Well-being

The results of a MANCOVA with repeated measures showed a significant effect of the covariate age. Following the univariate tests for the eight sub-dimensions, this effect reaches the level of significance only for the sub-dimension irritability, *F*(1,48) = 5.42, *p* = 0.024, $\eta_{p}^{2}$ = 0.101. All other effects do not reach the 5%-level of significance. The coefficients are shown in **Table 1**. Regarding the sub-dimension irritability, correlations between the age of the participants and their reported irritability at the end of the study sessions show that irritability tends to decrease with age [t1: *r* = -0.227, *p* = 0.109; t2: *r* = -0.233, *p* = 0.100; partial correlation (controlled variable: hearing impairment): t1: *r* = -0.286, *p* = 0.044; t2: *r* = -0.300, *p* = 0.035].

**Table 1:** Results of the ANCOVAs and MANCOVAs.

	*F*	*df*	*df_Error_*	*p*	$\eta_{p}^{2}$
**Subjective emotional well-being**					
Age	2.83	8	41	0.013	0.356
Hearing impairment	1.18	8	41	0.336	0.187
Time	1.08	8	41	0.396	0.174
Time × hearing impairment	0.51	8	41	0.842	0.091
**Subjective evaluation of perceived recovery**					
Age	0.10	1	48	0.752	0.002
Hearing impairment	0.11	1	48	0.747	0.002
Time	0.17	1	48	0.680	0.004
Time × hearing impairment	0.33	1	48	0.912	<0.001
**Subjective evaluation of perceived fatigue**					
Age	0.31	1	46	0.584	0.007
Hearing impairment	0.01	1	46	0.949	<0.001
Time	0.07	1	46	0.796	0.001
Time × hearing impairment	0.01	1	46	0.958	<0.001
**Subjective physical symptoms**					
Age	0.31	7	42	0.945	0.049
Hearing impairment	0.30	7	42	0.952	0.047
Time	1.00	7	42	0.444	0.143
Time × hearing impairment	1.77	7	42	0.120	0.227
**Cortisol level**					
Age	6.04	1	48	0.018	0.112
Hearing impairment	4.76	1	48	0.034	0.090
Time	0.50	1.06	51.03	0.496	0.010
Time × hearing impairment	3.52	1.06	51.03	0.064	0.068

### Subjective Evaluation of Perceived Recovery/Fatigue

Hearing impairment does not have a significant effect neither on the perceived recovery of the participants nor on their perceived fatigue at the end of the study sessions. Also, the covariate age does not significantly impact the results and all within-subject effects did not reach the 5%-level of significance. The coefficients are shown in **Table 1**.

### Subjective Physical Symptoms

The result of a MANCOVA with repeated measures showed no significant effect of hearing impairment for subjective physical symptoms. Also, the covariate age as well as effects of measurement time did not reach the 5%-level of significance (see **Table 1**).

### Cortisol Level

The results of an ANCOVA with repeated measures showed a significant effect for the covariate age and for hearing impairment. Furthermore, the effect time x hearing impairment just failed significance. The results of the analysis of covariance indicated that although age had a significant effect on the cortisol level, group differences remained significant. All other effects do not reach the 5%-level of significance (see **Table 1**). The descriptive statistics are shown in **Table 2**. Hearing impaired employees tend to show lower cortisol levels than normal hearing employees. The effect is most pronounced for the waking cortisol level of the second day (t2). Follow-up analyses (ANCOVAs) that were conducted for each time point, show that the effect only reaches the level of significance for this time of measurement (see **Table 3**). Hearing impaired employees show a significant lower waking cortisol level than normal hearing employees.

**Table 2:** Descriptive statistics: cortisol level – hearing impairment groups.

	Hearing impairment groups	*M*	*SD*	*N*
Cortisol t1 [nmol/l]	Normal hearing group	2.49	1.39	30
	Hearing impaired group	2.49	1.92	21
	Total	2.49	1.61	51
Cortisol t2 [nmol/l]	Normal hearing group	24.24	14.79	30
	Hearing impaired group	19.91	11.19	21
	Total	22.46	13.48	51
Cortisol t3 [nmol/l]	Normal hearing group	2.48	1.02	30
	Hearing impaired group	2.27	1.31	21
	Total	2.40	1.14	51

**Table 3:** Cortisol level – results of the ANCOVAs.

	*F*	*df*	*df_Error_*	*p*	$\eta_{p}^{2}$
**t1**					
Age	1.31	1	48	0.258	0.027
Hearing impairment	0.25	1	48	0.621	0.005
**t2**					
Age	4.68	1	48	0.035	0.089
Hearing impairment	4.00	1	48	0.051	0.077
**t3**					
Age	2.79	1	48	0.101	0.055
Hearing impairment	1.76	1	48	0.192	0.035

## Discussion

The aim of the study was to examine whether age and hearing impairment do have an impact on psychophysiological and subjective effects of long working hours. The results show that from a subjective point of view (subjective emotional well-being, subjective evaluation of perceived recovery/fatigue, subjective physical symptoms), no significant group differences can be shown in our study. Furthermore, with one exception for the sub-dimension irritability of the subjective emotional well-being, the covariate age does not have a significant impact on the subjective results. Regarding the sub-dimension irritability it can be shown that irritability tends to decrease with age. However, the findings of the current study do not support the previous research results that older employees seem to have a stronger need of recovery than younger employees (e.g., Jansen et al., 2002; Kiss et al., 2008). Furthermore, the findings are contrary to previous studies which have suggested a reduced subjective emotional well-being of hearing impaired employees (Monzani et al., 2008) and a higher need of recovery after work of people with poorer than of people with better hearing status (Nachtegaal et al., 2009). A possible explanation for these results may be that hearing impaired employees may have a variety of conscious and unconscious coping strategies to cope with demanding working situations. This is an important issue for future research. Furthermore, the motivation of the participants during the study was not assessed. It could be argued that the different groups do differ in their motivation. Maybe older and/or hearing impaired participants do not have to prove themselves as strongly as others. It may be that these participants have perceived the study less demanding than others. The result that the irritability of the participants at the end of the study sessions tends to decrease with age can also be seen as indication for this. Therefore, participant’s motivation should be included in future studies.

Interestingly, data of salivary cortisol reveal that the non-significant subjective results are not supported by the objective physiological saliva cortisol data. A significant effect of hearing impairment was shown for the cortisol level. Furthermore, the effect time x hearing impairment just failed significance and a significant effect for the covariate age can be reported. The results of the analyses indicated that although the covariate age had a significant effect on the cortisol level, group differences remained significant: Hearing impaired employees tend to show lower cortisol levels than normal hearing employees. The effect is most pronounced for the waking cortisol level of the second day (t2). Follow-up analyses that were conducted for each time point showed that the effect only reaches the level of significance for this time of measurement. Hearing impaired employees show a significant lower waking cortisol level than normal hearing employees. Following Boucsein and Backs (2000) an increase of cortisol level is an indicator for mental and emotional strain. Following these results, long working hours seem to have a psychophysiological impact on normal hearing employees. The comparison of normal hearing and hearing impaired employees shows that at least three hours of experimentally induced longer working hours result in a stronger response of the stress system of normal hearing employees in the morning of the following day. This effect differs from the expectation that the effort of a hearing impaired participant must expend in order to receive the same perceptual performance as a normal hearing employee (effortfulness hypothesis; Rabbitt, 1968; Kahneman, 1973; McCoy et al., 2005). This might also have an impact on psychophysiological reactions of the organism measured with saliva cortisol. To exclude an effect of possible confounding variables on the waking cortisol level, group differences regarding variables such as awakening time, sleep duration and perceived sleep quality were analyzed (e.g., Edwards et al., 2001). The results show that within our sample, normal hearing and hearing impaired participants do not differ significantly within these variables.

Furthermore, the idea arises that following Steptoe et al. (1998, 2000) which emphasize that job strain is able to influence the cortisol level, lower cortisol levels of hearing impaired employees compared to normal hearing employees (esp. at t2) may also be explained by different levels of job strain of the participants. Unfortunately, this variable was not included in the study but the profession of the participants was surveyed. Additionally they were asked if they have a leadership position which may also correlate with subjectively experienced job strain. Regarding the two hearing impairment groups, no significant differences can be shown concerning the professions of the participants. Also, they do not differ significantly with regard to leadership positions. Another explanation that is not proven yet is that hearing impaired employees anyway have to struggle with a lot of impairment-related difficulties during their normal working life and therefore the additional expense of some additional working hours has not that much negative influence on them at the first sight. It is therefore likely that normal hearing employees are more affected by long working hours than their hearing impaired colleagues, especially after the first day of their occurrence. Another explanation of the results may be that especially persons with mild hearing impairment, who were often part of samples with persons of working age, are able to compensate their impairment and adapt to the situation quite well. Further studies should investigate whether the effect can be confirmed for extended periods of long working hours which might be more demanding than 2 days with overtime hours.

## Study Limitations and Directions for Future Research

This study has some limitations. First, our study participants had to work long working hours on two consecutive days. Especially regarding the non-significant subjective effects but also regarding effects on the cortisol level (see also Marchand et al., 2012), it is supposed that this singular overtime event was maybe not severe enough to make possible subjective effects of long working hours visible. Further studies should therefore address extended periods of long working hours to receive more information.

Second, we did not test our participants on “normal” working days without working overtime hours. We therefore recommend that future research shall include days without extended working hours as additional control condition. Other possibilities to enable a comparison between normal and long working hours are the inclusion of a baseline measurement of cortisol level before the sessions of additional work and/or the inclusion of a control group working normal working hours, into the study design.

One limitation of the study is that young/old comparisons in psychophysiological parameters can always be questioned, as the physiological system and the systems reactivity normally change with increasing age (Janke and Kallus, 1995).

Furthermore, from a practical point of view it would be interesting to examine possible demanding working situations in future studies that are more and more typical for a global working world, like audio-conferences during unusual working hours (e.g., late in the evening, very early in the morning) and/or in foreign languages. Another facet with practical implications is the impact of environmental conditions of the workplace like noise or lack of space or privacy like it could be found in open-plan offices. In addition, the investigation of different categories of work (e.g., professionals, white-collar workers, blue-collar workers) should be addressed in future studies.

## Conclusion

The results of our study show no significant effects for age and hearing impairment on the intensity of subjective consequences of long working hours. But, age and hearing impairment do matter from a psychophysiological point of view. Psychophysiological responses (saliva cortisol level) on long working hours differ significantly between hearing impaired and normal hearing employees. Interestingly, the results suggest that long working hours were more demanding for normal hearing than for hearing impaired employees. Furthermore, normal hearing employees tend to show a higher waking cortisol level after 1 day with long working hours than hearing impaired employees. To uncover possible long-term effects further research is still required.

## Ethics Statement

This study was carried out in accordance with the recommendations of the ethics committee of the University of Graz with written informed consent from all subjects. The study was approved by the ethics committee of the University of Graz.

## Author Contributions

All authors listed have made a substantial, direct and intellectual contribution to the work, and approved it for publication.

## Conflict of Interest Statement

The authors declare that the research was conducted in the absence of any commercial or financial relationships that could be construed as a potential conflict of interest.
